# Supplementary figures and images for: Identification of Genetic Alterations in Rapid Progressive Glioblastoma by Use of Whole Exome Sequencing
Source: Diagnostics (Basel). 2023 Mar 7;13(6):1017. doi: 10.3390/diagnostics13061017 (PMC10047503; doi:10.3390/diagnostics13061017)

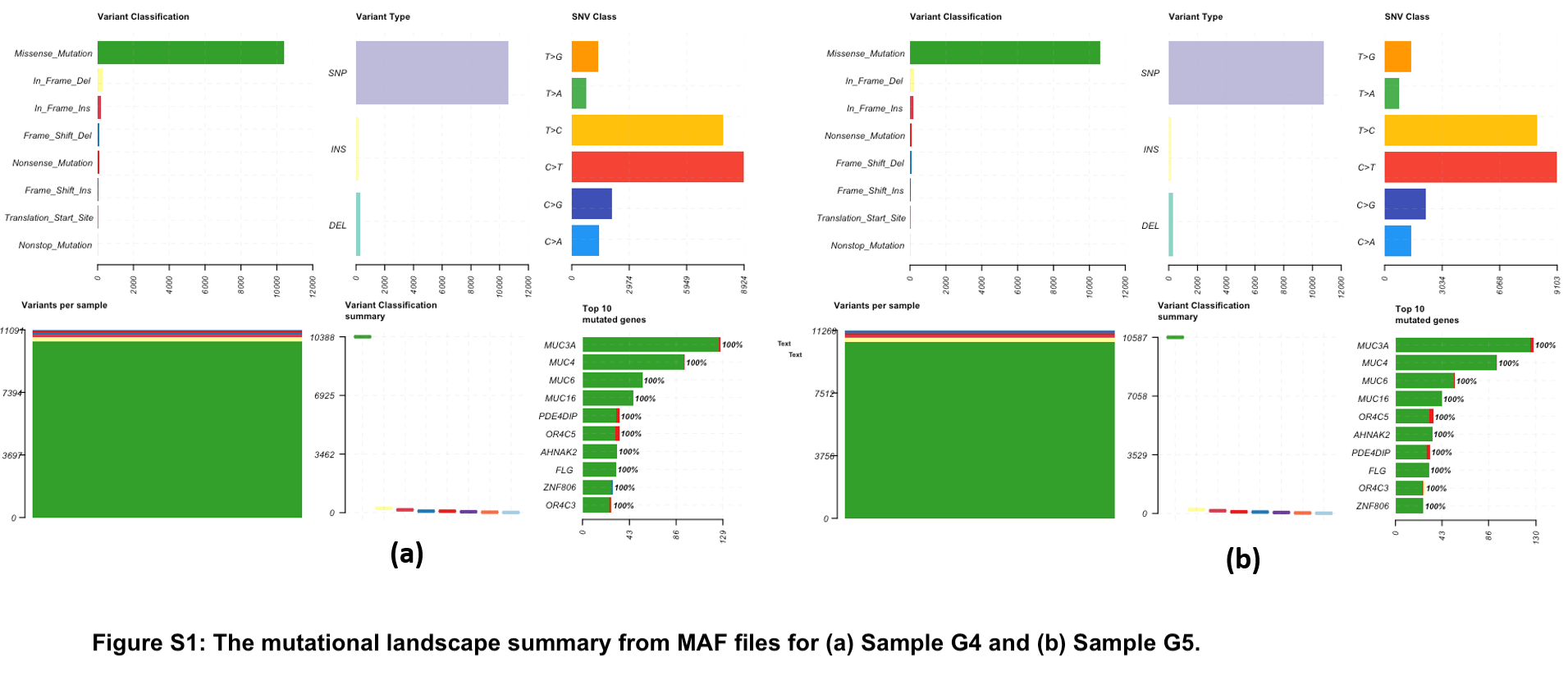

Supplement: Supplementary file 1 [file diagnostics-13-01017-s001.zip › Supplementary Figure S1.png]

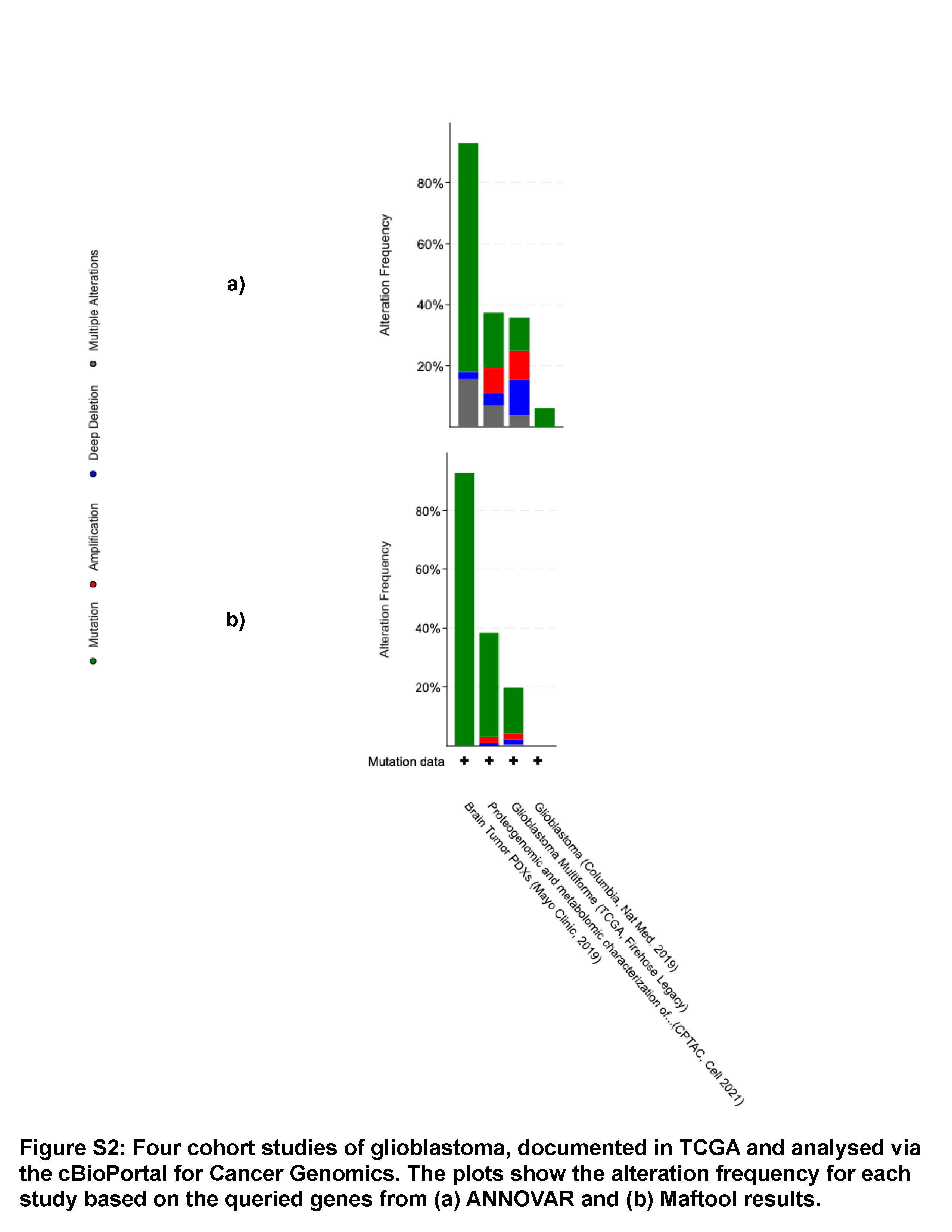

Supplement: Supplementary file 1 [file diagnostics-13-01017-s001.zip › Supplementary Figure S2.png]
